# Acupuncture as add-on treatment for functional dyspepsia: A protocol for systematic review

**DOI:** 10.1097/MD.0000000000024403

**Published:** 2021-02-19

**Authors:** Chan-Young Kwon, Seok-Jae Ko, Boram Lee, Jae Myung Cha, Jae-Woo Park

**Affiliations:** a Department of Oriental Neuropsychiatry, College of Korean Medicine, Dong-eui University, Busan; b Department of Gastroenterology, College of Korean Medicine, Kyung Hee University, Seoul; c Clinical Medicine Division, Korea Institute of Oriental Medicine, Daejeon; d Department of Internal Medicine, Kyung Hee University School of Medicine, Seoul, Republic of Korea.

**Keywords:** acupuncture, dyspepsia, functional dyspepsia, protocol, systematic review

## Abstract

**Background:**

Functional dyspepsia (FD) is a common functional gastrointestinal disease with a high prevalence. However, due to the limitations of conventional Western treatments, such as acid suppressants, prokinetics, Helicobacter pylori eradication treatment, and antidepressants, the popularity of complementary and alternative medicine, such as acupuncture, is steadily increasing. We describe the methods that will be used to evaluate the effectiveness and safety of acupuncture as add-on therapies to conventional Western medications in patients with FD.

**Methods and analysis:**

A total of 12 English, Korean, and Chinese electronic databases will be searched by 2 researchers from their inception dates to December 2020. Randomized controlled trials assessing the effectiveness and safety of acupuncture as add-on therapies to conventional Western medications in patients with FD will be included. The primary outcome measure will be the symptom score of FD, and secondary outcome measures will be total effective rate, quality of life, level of gut peptide hormones, incidence of adverse events, and recurrence rate. Data analysis will be performed using the Review Manager version 5.3. The risk of bias of the included studies and the quality of evidence for the main findings will be evaluated using the Cochrane Collaborations risk of bias tool and the Grading of Recommendations Assessment, Development, and Evaluation approach, respectively.

**Conclusion:**

The findings of this review will provide evidence on the complementary effectiveness and safety of acupuncture for FD for clinicians, patients, and policy makers in decision making.

**OSF registration number:**

DOI 10.17605/OSF.IO/MXREN (https://osf.io/mxren).

## 1 Introduction

Functional dyspepsia (FD) is a common functional gastrointestinal disease and can be defined as “postprandial fullness, early satiation, epigastric pain, or epigastric burning, which are unexplained after a routine clinical evaluation”.^[1]^ Although the prevalence rate is slightly higher in Western countries, FD affects many individuals globally (9.8%–20.2% in Western and 5.3%–12.8% in Eastern countries, based on the ROME III criteria).^[2]^ This disease by itself does not cause fatal outcomes for patients; however, it is a cause of serious impact on the quality of life (QoL) and work productivity of patients, resulting in a severe social and economic burden.^[3,4]^ Today, FD is generally divided into 2 subcategories: postprandial distress syndrome and epigastric pain syndrome.^[1]^ The etiology of FD is considered to be multifactorial, and several factors are considered to be related, including motility disturbances, sensorimotor disorders, visceral hypersensitivity, postinfectious plasticity of the duodenum, elevated mucosal permeability, some biopsychosocial factors, and disturbances of the autonomic and enteric nervous system.^[5]^ Conventional medicine approaches that can be used from the perspective of evidence-based medicine (EBM) include proton pump inhibitors, Helicobacter pylori eradication treatment, antidepressants, and psychotherapy.^[5]^ However, due to the limitations of these conventional treatments, the popularity of complementary and alternative medicine (CAM) is steadily increasing.^[6]^

Currently, popular CAM modalities for FD include herbal supplements, acupuncture, and hypnosis.^[6]^ In particular, since acupuncture is a non-pharmacological intervention, it has the advantage of being free from herb-drug interactions with a high potential for use as an adjuvant therapy along with conventional medications. In addition, according to a comprehensive recent review on acupuncture, it may lead to clinical improvement of FD concerning the regulation of gastric motility, gastric accommodation, mental status, gastrointestinal hormones, and central and autonomic functions.^[7]^ Although several previously published systematic reviews (SRs) suggest the potential clinical benefits of acupuncture,^[7–11]^ to date, no SR has comprehensively reviewed the effectiveness of acupuncture as an add-on therapy to conventional medication for FD and critically evaluated the quality of evidence using methodologies such as the Grading of Recommendations, Assessment, Development, and Evaluation (GRADE).^[12]^ This approach can help to make potential recommendations for decision making by clinicians, patients, and policy makers.^[13]^

In this study, we will attempt to critically evaluate the effectiveness and safety of acupuncture as an add-on treatment to conventional Western medications for FD as well as the quality of evidence from the findings. This review will provide reliable evidence and consequently confirm the complementary role of acupuncture from the perspective of EBM, helping patients and healthcare providers in shared decision making, and policy makers to relieve the enormous economic and social burden caused by FD.

## 2 Methods

### 2.1 Study registration

This protocol has been registered on the OSF registries (URL: https://osf.io/mxren). If there are any changes to this protocol, we will report the dates, changes, and rationales on the Comments section in OSF registries. We reported the protocol according to the Preferred Reporting Items for Systematic Review and Meta-Analysis (PRISMA)-Protocols 2015 statement.^[14]^

### 2.2 Data sources and search strategy

Two researchers (CYK and BL) will independently search the following electronic databases from their inception dates to December 2020: 5 English databases (Medline via PubMed, EMBASE via Elsevier, Cochrane Central Register of Controlled Trials, Allied and Complementary Medicine Database via EBSCO, and Cumulative Index to Nursing and Allied Health Literature via EBSCO), 5 Korean databases (Oriental Medicine Advanced Searching Integrated System, Korean Studies Information Service System, Research Information Service System, Korean Medical Database, and Korea Citation Index), and 2 Chinese databases (China National Knowledge Infrastructure and Wanfang data). The reference lists of the relevant articles and trial registries, such as clinicaltrials.gov, will be manually searched to identify additional gray literature. We set up a search strategy through sufficient discussion with experts in FD and SR methodology. The search strategy in Medline is shown in Table 1, which will be modified and used in other databases.

**Table 1:** Search strategies for the Medline via PubMed.

#1. indigestion∗
#2. intestin∗ OR digest∗ OR gastr∗ OR gut OR epigastr∗ OR stomach∗
#3. #1 AND #2
#4. dyspepsia∗
#5. Epigastric [TIAB] AND pain [TIAB]
#6. Epigastric [TIAB] AND burn∗ [TIAB]
#7. Rome∗ AND criteria∗
#8. (disturbance∗ OR disorder∗ OR difficult∗ OR dysfunction∗ OR disease∗ OR impair∗ OR condition∗ OR abnormal∗ OR illness∗ OR patholog∗ OR discomfort∗ OR hazard∗ OR damage∗ OR injur∗ OR irritab∗ OR pain∗ OR distress∗ OR burning) AND postprandial∗
#9. #3 OR #4 OR #5 OR #6 OR #7 OR #8
#10. Acupuncture [MH] OR “Acupuncture Therapy” [MH] OR “Acupuncture Points” [MH] OR acupunct∗ [TIAB] OR Electroacupuncture [MH] OR electroacupunct∗ [TIAB] or electro-acupunct∗ [TIAB] OR Auriculotherapy [MH] OR “ear acupuncture” [TIAB] OR acupoint [TIAB] OR Acupressure [MH] OR acupressure [TIAB]
#11. “Randomized Controlled Trial” [PT] OR “Controlled Clinical Trial” [PT] OR randomized [TIAB] OR placebo[TIAB] OR “Clinical Trials as Topic” [Mesh: noexp] OR randomly [TIAB] OR trial [TI]
#12. Animals [MH] NOT humans [MH]
#13. #9 AND #10 AND #11 NOT #12

### 2.3 Inclusion criteria

#### 2.3.1 Types of studies

We will include randomized controlled trials (RCTs) assessing the effectiveness and safety of acupuncture as an add-on treatment to conventional Western medications for FD. We will include only parallel-group studies and exclude crossover designs for lowering the potential risk of bias. There will be no restrictions on the publication language and publication status.

#### 2.3.2 Types of participants

Studies involving patients with FD diagnosed using standardized diagnostic criteria such as ROME or clinical symptoms will be included, regardless of sex, age, or race. Studies involving patients with organic causes of dyspepsia will be excluded.

#### 2.3.3 Types of interventions

Studies involving any type of acupuncture (including manual acupuncture, electroacupuncture, auriculotherapy, and acupressure) as add-on therapies to conventional Western medication for FD such as acid suppressants, prokinetics, Helicobacter pylori eradication, fundic relaxants, or antidepressants as treatment interventions will be included. As the control intervention, studies involving conventional Western medication for FD, such as acid suppressants, prokinetics, Helicobacter pylori eradication, fundic relaxants, or antidepressants, will be included. We will exclude studies that used herbal medicine as a treatment or control group.

#### 2.3.4 Types of outcome measures

The primary outcome measure will be the symptom score of FD, measured using the Nepean Dyspepsia Index,^[15]^ Gastrointestinal Symptom Rating Scale,^[16]^ Dyspepsia Symptom Severity Index,^[17]^ and visual analog scale.

The secondary outcome measures will be (a) total effective rate; (b) QoL measured by the FD-QoL^[18]^ and the short-form health survey^[19]^; (c) level of gut peptide hormones such as motilin, ghrelin, and gastrin; (d) incidence of adverse events during the treatment period; and (e) recurrence rate.

### 2.4 Study selection and data extraction

**Figure 1:**
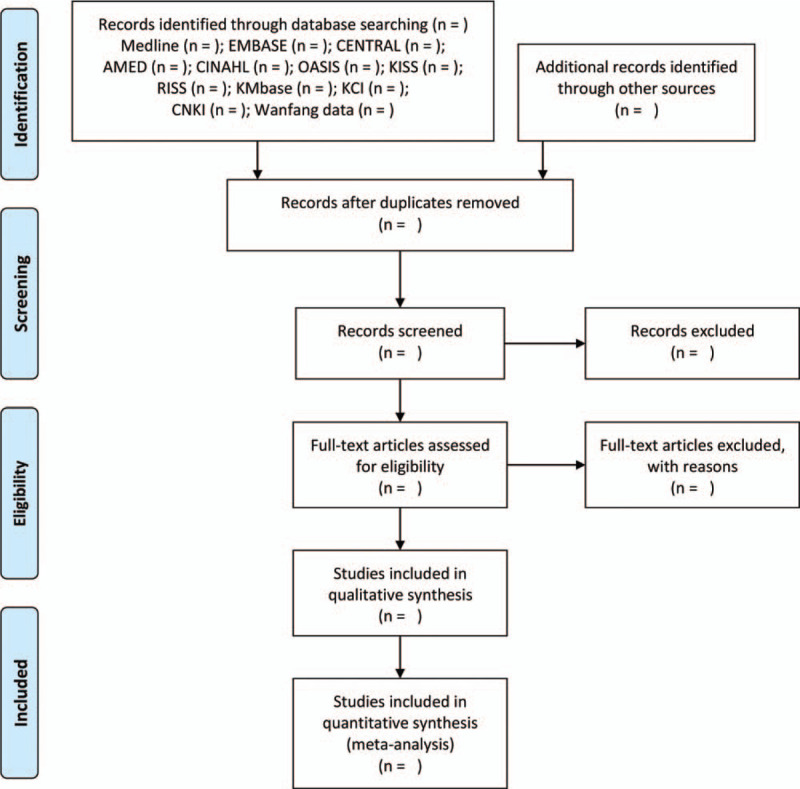
A PRISMA flow diagram of the literature screening and selection processes. AMED = Allied and Complementary Medicine Database, CENTRAL = Cochrane Central Register of Controlled Trials, CINAHL = Cumulative Index to Nursing and Allied Health Literature, CNKI = China National Knowledge Infrastructure, KCI = Korea Citation Index, KISS = Koreanstudies Information Service System, KMbase = Korean Medical Database, OASIS = Oriental Medicine Advanced Searching Integrated System, RISS = Research Information Service System.

First, 2 researchers (CYK and BL) will remove duplicate documents from studies retrieved from the database and other sources and will examine the eligibility by reviewing titles and abstracts using EndNote X8 (Clarivate Analytics, Philadelphia, USA). In addition, the possibility of final inclusion will be reviewed by examining the full text for suitable literature. The study selection process will be presented using the PRISMA flow diagram (Fig. 1).

We will use a standardized, pre-defined, pilot-tested Excel form to extract relevant data from the included studies. The following information will be extracted by 2 researchers (CYK and BL) independently: the first authors name, year of publication, country, sample size and dropout, details of participants, treatment and control intervention, duration of intervention, outcome measures, adverse events, information for the assessment of the risk of bias, and funding sources. When the data are insufficient or ambiguous, we will contact the corresponding authors of the included studies via e-mail. We will resolve any disagreements in the study selection and data extraction process via discussion with other researchers (SJK).

### 2.5 Quality assessment

We will assess the methodological quality of the included RCTs using the Cochrane Collaborations risk of bias tool.

The following 7 domains will be evaluated independently by 2 researchers (CYK and BL): random sequence generation, allocation concealment, blinding of participants and personnel, blinding of outcome assessors, completeness of outcome data, selective reporting, and other biases. In particular, we will assess the other bias domains based on the statistical homogeneity of baseline clinical characteristics such as mean age, sex, or disease severity between the treatment and control groups. We will evaluate each of the 7 domains as “low risk,” “unclear risk,” or “high risk”.^[20]^

The two researchers (CYK and BL) will also assess the quality of evidence on the main findings using the GRADE approach (https://gradepro.org/).^[12]^ The risk of bias, inconsistency, indirectness, imprecision of the results, and publication bias will be assessed and the quality of evidence of the main findings will be presented as “very low”, “low”, “moderate”, or “high” Any discrepancies will be resolved via discussion with another researcher (SJK).

### 2.6 Data analysis

We will present the demographic characteristics of the participants, details of the treatment and control interventions, and outcomes from all included studies. We will quantitatively synthesize the studies using the same type of treatment and control intervention, with the same outcome measures, using Review Manager software (version 5.3; Cochrane, London, UK). We will present the continuous outcomes using the mean difference and binary outcomes using the risk ratio, with 95% confidence intervals.

Heterogeneity between the studies in the meta-analysis will be assessed using both the χ^2^ test and the *I*^2^ statistic, and we will consider an *I*^2^ value >50% to be indicative of substantial heterogeneity and an *I*^2^ value >75% to be indicative of considerable heterogeneity. The results will be pooled using a random-effects model if the included studies have significant heterogeneity (*I*^2^ value >50%), while a fixed-effects model will be used if the heterogeneity is not significant. We will also use a fixed-effects model if the number of studies included in the meta-analysis is less than 5 owing to the lack of precision of the estimate of the between-study variance.^[21]^

If the necessary data are available, we will conduct a subgroup analysis according to the following criteria: (a) type of conventional medication (acid suppressants, prokinetics, Helicobacter pylori eradication, fundic relaxants, or antidepressants), and (b) type of acupuncture (manual acupuncture, electroacupuncture, auriculotherapy, or acupressure). We will perform sensitivity analyses to identify the robustness of the results of the meta-analysis by excluding (a) studies with a high risk of bias and (b) outliers that are numerically distant from the rest of the data. Furthermore, we will also assess evidence of publication bias using funnel plots if more than ten studies are included in the meta-analysis.

### 2.7 Ethics and dissemination

Ethical approval is not required since this is a protocol for a SR. The results of this SR will be disseminated by publication in a peer-reviewed journal or presentation at relevant conferences.

## 3 Discussion

Being a common functional gastrointestinal disorder, FD not only seriously impairs the QoL of patients with FD but also causes serious economic and social burden.^[3,4]^ Conventional medical approaches alone do not have high patient satisfaction, and many patients with FD pursue CAM treatment.^[22]^ Acupuncture is one of the most studied CAM modalities for FD,^[7–11]^ and the quality of evidence from comprehensive evidence still needs to be clearly and critically assessed. Therefore, in this review, the complementary role of acupuncture in FD, i.e., the evidence for its effectiveness and safety as an add-on therapy to conventional Western medications for FD as well as the quality of evidence from the findings will be critically evaluated. The findings of this review will help patients and healthcare providers to facilitate shared decision making and policy makers reduce the socioeconomic burden caused by this disease through policy making.

## Author contributions

**Conceptualization:** Seok-Jae Ko, Jae-Woo Park.

**Funding acquisition:** Jae-Woo Park.

**Methodology:** Chan-Young Kwon, Seok-Jae Ko, Boram Lee.

**Supervision:** Jae Myung Cha, Jae-Woo Park.

**Writing – original draft:** Chan-Young Kwon, Boram Lee.

**Writing – review & editing:** Seok-Jae Ko, Jae Myung Cha, Jae-Woo Park.

Supplemental Digital Content: Supplement 1. PRISMA-P 2015 Checklists

## Supplementary Material

Supplemental Digital Content
